# Photoactivatable Aptamer-Based Biosensors for Point-of-Care Testing: Advances and Applications

**DOI:** 10.3390/bios15060336

**Published:** 2025-05-24

**Authors:** Siyuan Wang, Xinyun Cui, Zixuan Zhong, Jingjing Zhang

**Affiliations:** 1 State Key Laboratory of Analytical Chemistry for Life Science, School of Chemistry and Chemical Engineering, Chemistry and Biomedicine Innovation Center (ChemBIC), Nanjing University, Nanjing 210023, China; 2 School of Life Sciences, Nanjing University, Nanjing 210023, China

**Keywords:** photoactivatable, aptamers, biosensors, point-of-care testing (POCT)

## Abstract

Photoactivatable aptamer sensing technology is widely used in various detection fields due to its precise spatiotemporal regulation ability, flexible material compatibility, and excellent detection performance. By introducing an optical response mechanism to regulate the efficient recognition of the target by the sensor, this strategy further broadens the regulation means of the aptamer. The application of photoactivated aptamer biosensors in point-of-care testing (POCT) can significantly improve the selectivity, sensitivity, and dynamic response ability of the POCT system. This review systematically explores the design principle and regulation mechanism of photoactivatable aptamers, with a focus on reviewing the application progress of them in the POCT platform. In addition, the existing challenges and future development trends are also discussed. It is expected that this biosensor based on photoactivatable aptamers will continue to drive POCT towards higher sensitivity, intelligence, and scene adaptability, providing innovative tools for precision medicine and environmental health monitoring.

## 1. Introduction

The advancement of precision medicine has driven an urgent demand for diagnostic technologies that simultaneously achieve rapid analysis, high sensitivity, and portability [1,2,3]. Traditional antibodies have dominated clinical diagnosis as molecular recognition elements for many years, but their deficiencies in production cost, stability, batch consistency, etc., have limited their application in certain scenarios. Since their first report in the early 1990s [4,5], aptamers have rapidly become an ideal alternative to antibodies due to their unique physicochemical properties [6,7,8]. Aptamers are a class of single-stranded sequences composed of RNA, DNA, or artificially synthesized nucleic acid molecules [9,10], which can bind to target molecules (including small molecules [11,12,13], proteins [14,15,16,17,18], viruses [19,20,21], cells [22,23,24], and even tissues [25,26,27]) with high affinity and specificity through specific three-dimensional conformations [28,29,30]. Compared with antibodies, aptamers exhibit advantages in terms of size, chemical modifiability, and stability [31,32]. These characteristics have drawn much attention to the application of aptamers in biosensors [33,34,35,36], especially demonstrating greater flexibility and plasticity in meeting the sensing requirements in harsh environments [37,38] and portable devices [39,40,41,42]. In recent years, researchers have further developed stimulus-responsive aptamer sensors, which have advantages such as high specificity, high sensitivity, and precise dynamic regulation capabilities [43,44,45]. Among them, “photoactivatable aptamers” that regulate aptamers through photosensitive domains or light response mechanisms have become a highly promising research direction [46,47,48,49,50].

Photoactivatable aptamers achieve precise spatiotemporal control of target molecules by introducing photoresponsive chemical groups or constructing photosensitive nucleic acid structures, causing reversible or irreversible changes in their recognition ability or conformation under light of specific wavelengths [51,52,53,54,55,56,57,58]. This strategy not only enhances the dynamic regulation ability of aptamer function, but also endows the sensing system with higher selectivity and background suppression ability [59,60].

In practical applications, the demand for portable point-of-care testing (POCT) technology is increasing day by day [61,62]. POCT emphasizes the miniaturization of equipment, the rapidity of detection, the simplicity of operation, and the feasibility of on-site application [63,64,65]. It is particularly suitable for resource-limited areas, emergency medicine, and home health monitoring [66,67]. Combining the aptamer with POCT can construct a biosensing platform with high sensitivity and high specificity [68,69]. In recent years, aptamer sensors that integrate technologies such as smart phones [70], microfluidic chips [71,72,73], and nanomaterials [74] have been continuously reported, demonstrating excellent performance in detecting heavy metals, bacteria, viruses, and biomarkers [75,76,77,78]. Furthermore, the aptamer sensor empowered by the optical activation mechanism is expected to enhance the controllability and detection accuracy of POCT devices, making them not only suitable for fixed-point detection but also capable of intelligent response and signal amplification.

**Figure 1:**
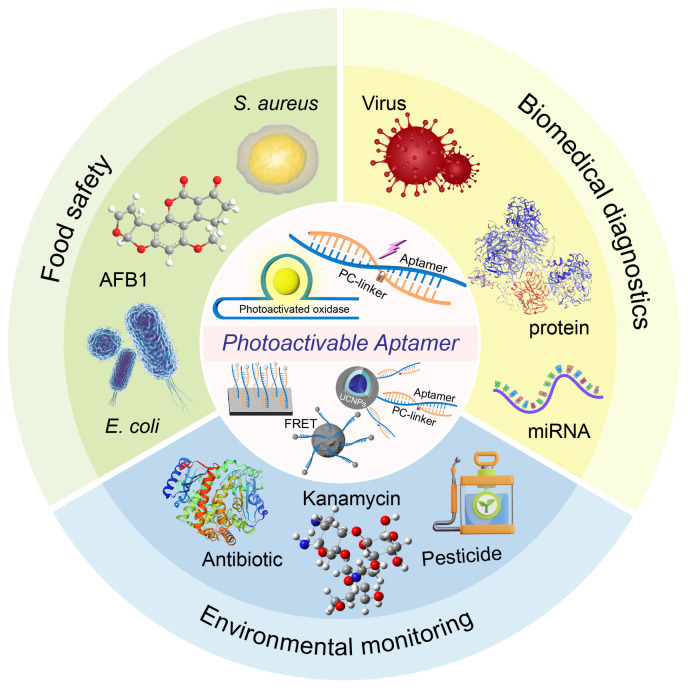
Overview of aptamer-based photoactivated biosensors for POCT.

In summary, aptamers, with their excellent molecular recognition capabilities and engineering modification potential, are gradually becoming key biometric components in POCT platforms [79,80]. This review systematically introduces the design principle qne regulation mechanism of photoactivatable aptamers and their application progress in POCT, and looks forward to their future development potential in precision diagnosis (Figure 1).

## 2. The Mechanism of Action of Photoactivatable Aptamers

Aptamers can be used to construct various photoactivatable POCT platforms with high sensitivity and specificity by introducing photosensitive linkers and combining them with quantum dots or other nanomaterials.

### 2.1. Optical Control of Aptamer

**Figure 2:**
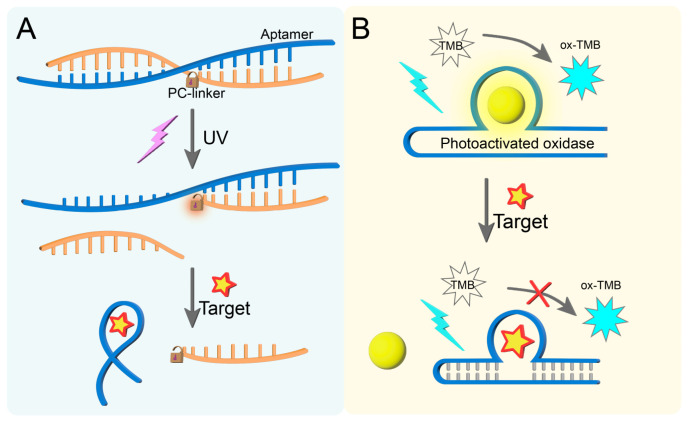
(**A**) Photoactivated aptamers based on the action of PC-linker. (**B**) Photoactivated aptamers based on the action of photoactivated oxidase.

A photocleavable linker (PC-linker) is a type of chemical group that can undergo irreversible cleavage under ultraviolet (UV) light (typically 365 nm) irradiation. Its core structure contains o-nitrobenzyl or coumarin derivatives and is covalently linked to the aptamer through ester bonds or amino bonds [81,82]. For instance, Jia et al. [83] exploited the light-cleavable characteristic of a PC-linker by covalently attaching it to a complementary DNA strand capable of hybridizing with the aptamer sequence through Watson–Crick base pairing. This strategic modification creates a sterically hindered conformation that physically blocks aptamer-target recognition in the absence of light irradiation (Figure 2A). Only after the PC-linker is broken by UV light irradiation can the aptamer combine with the target to generate the output signal.

Photoactivated oxidase is an oxidase produced through photoactivation, usually copolymerized from dicyandiamide and barbituric acid [84,85]. This enzyme can catalyze oxidation under visible light to produce a green product. Thioflavin T(ThT) has a unique photoactivated oxidase activity. Therefore, Lu et al. [86] achieved the fluorescence colorimetric detection of estradiol by binding ThT to the cavity site of the estradiol aptamer (Figure 2B). ThT and the aptamer complex can oxidize the colorless TMB substrate to its oxide ox-TMB under 460 nm light exposure, generating a colorimetric signal. In the presence of the target, the specific binding between the aptamer and the target causes ThT to fall off and the signal to weaken. Isoquinoline alkaloids are also a kind of photoactivated oxidase, which show significant photoactivated oxidation capacity after binding to the aptamer sequence. The aptamer–berberine complex can oxidize all substrates under light to show obvious colorimetric changes. In the presence of the target, the aptamer recognizes the target, and the berberine dissociation cannot oxidize the substrate, resulting in a weakened colorimetric signal. Therefore, Lu et al. [87] used the characteristics of isoquinoline alkaloids and combined them with different aptamers to detect different targets under light conditions.

### 2.2. Optical Control of Materials

**Figure 3:**
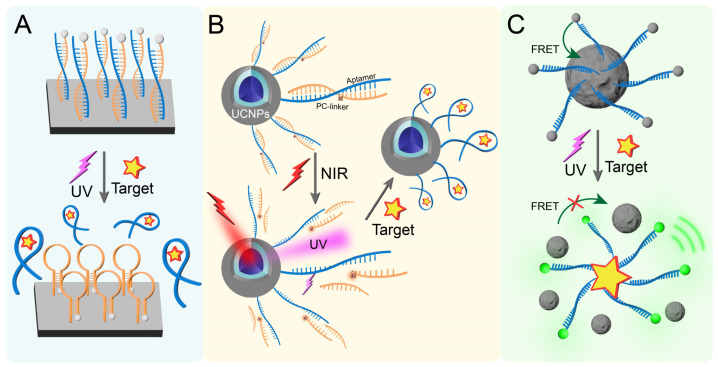
(**A**) Schematic diagram of photoactivated aptamers based on semiconductor materials. (**B**) Illustration of NIR light-activated aptamer sensing based on UCNPs and PC-linker combination. (**C**) Photoactivated sensing based on the FRET effect of PDANS.

Semiconductor materials have unique photoelectric properties. When combined with aptamers, they can be used to construct a POCT detection platform for photoactivatable aptamers [88,89]. For example, an aptamer-based photoelectrochemical sensor can be developed by immobilizing thiol-modified aptamers on an electrode surface via Au-S coordination and hybridizing complementary DNA strands through base pairing (Figure 3A). Upon target binding under UV light irradiation, conformational changes disrupt the DNA duplex, releasing signal reporters and cDNA from the electrode to induce photocurrent reduction. In another case, Wu et al. [90] described a composite material system wherein cDNA is immobilized on the CeO_2_@MnO_2_ heterojunction, enabling site-specific molecular interactions through electrostatic anchoring. Due to the unique hairpin structure of cDNA, CeO_2_@MnO_2_ on cDNA can approach the ITO surface infinitely, generating a photocurrent response under visible light irradiation. cDNA can complement and pair with the target aptamer to form a double-stranded rigid structure, reducing the photocurrent. In the presence of the target, the aptamer specifically binds to the target and leaves the sensor surface. The cDNA will return to the hairpin structure, resulting in an increase in photocurrent and generating a signal.

Because upconversion nanoparticles (UCNPs) can absorb near-infrared (NIR) light and convert it into UV light and visible light, UCNPs have been widely used in the light sensors of near-infrared light-activated corresponding systems [91,92,93]. Combining the aptamer with UCNPs can construct an aptamer sensor activated by NIR light. As shown in Figure 3B, Zhang et al. [94] covalently coupled the aptamer hybrid chain modified with a PC-linker to the surface of UCNPs. Under NIR light irradiation, UCNPs can absorb photons and convert them into ultraviolet light, thereby breaking the PC-linker of the aptamer. In the presence of the target, the aptamer binds and recognizes the target.

Another strategy involves intelligent nanomaterials utilizing fluorescence resonance energy transfer (FRET) [95]. For instance, Ye et al. [96] designed and synthesized an aptamer-based nanoprobe, which is composed of polydopamine nanospheres (PDANSs) as energy receptors and aptamers (FAM-Apt) modified with the energy donor FAM through π-π stacking interactions (Figure 3C). In the presence of the target, the specific binding of the target to the aptamer will reduce the π-π stacking interaction, thereby releasing free FAM-Apt, which can produce significant green fluorescence under UV light excitation. Moreover, under the irradiation of 808 nm laser, this nanoprobe can produce photothermal effects.

## 3. The Application of POCT Based on Photoactivatable Aptamers in Different Fields

In the field of modern analytical testing, POCT has attracted much attention due to its advantages of rapidity, convenience, and on-site presence. As an emerging recognition molecule, photoactivatable aptamers have shown great potential in POCT applications in fields such as food safety, environmental monitoring, and biomedical diagnostics, thanks to their unique photoresponse characteristics and high specificity. We have summarized the detection targets, detection methods, optical control strategies and detection limits of photoactivatable aptamer POCT in different fields, as shown in Table 1.

### 3.1. Food Safety

In food safety testing, expeditious and accurate identification and quantification of hazardous substances is the key to protect public health. There are various types of contaminants in food, such as *Escherichia coli* (*E. coli*) [98], *Staphylococcus aureus* (*S. aureus*) [88,119,120], *Salmonella typhimurium* (*S. typhimurium*), AFB1, other bacteria [121] and biological toxins [101,102]. Although traditional detection methods (high-performance liquid chromatography, gas chromatography, mass spectrometry, etc.) can accurately detect them, these methods have disadvantages such as complex pretreatment, long detection time, and expensive equipment. Therefore, it is difficult to meet the demand for on-site rapid detection. Based on this, researchers have developed a series of POCT based on photoactivatable aptamers for the field of food safety.

**Figure 4:**
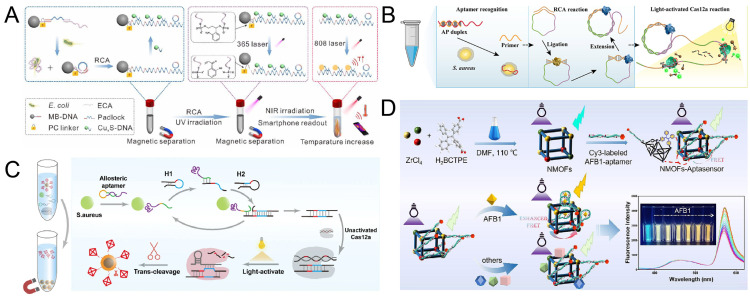
(**A**) Schematic of RCA-amplified photothermal biosensor for detection of *E. coli.* Reproduced with permission from ref. [98]. Copyright 2022, American Chemical Society. (**B**) The scheme of light-activated RCA-Cas12a method for *S. aureus* detection. Reproduced with permission from ref. [97]. Copyright 2023, Elsevier. (**C**) The scheme of the photoactivated CHA/Cas12a-based SERS platform for detecting *S. aureus.* Reproduced with permission from ref. [122]. Copyright 2023, Elsevier. (**D**) The scheme of the NMOFs-aptasensor for sensitive detection of AFB1. Reproduced with permission from ref. [123]. Copyright 2025, Springer Nature.

For example, Zhang et al. [98] constructed a dynamic and reversible bacterial detection system by integrating photoresponsive magnetic beads, rolling ring amplification (RCA), and photothermal sensing technology (Figure 4A). In this system, aptamers specifically recognizing *E. coli* are fixed on the surface of magnetic beads. The binding of the target bacteria triggers the RCA reaction, generating a large number of DNA products with repetitive sequences. Subsequently, ultraviolet light irradiation causes the photoresponsive DNA to break and release RCA products. These products hybridize with near-infrared excited Cu_x_S-modified DNA probes to enhance the photothermal signal. Through thermal imaging analysis on smart phones, the visual detection of *E. coli* was achieved, with a detection limit as low as 1.8 CFU/mL.

Based on this light-controlled dynamic interface, the optimization of the signal amplification strategy has become the key to improving the detection performance. As shown in Figure 4B, Wu et al. [97] designed a CRISPR/Cas12a system triggered by light-controlled crRNA and achieved dual-signal amplification in combination with RCA. The DNA of the target bacteria triggers RCA to generate repetitive sequences, which serve as the activation template for CRISPR/Cas12a. The crRNA modified by the photosensitive protective group releases active crRNA under UV light, activates the cleavage activity of Cas12a, and generates a fluorescence signal. This method can achieve rapid detection of *S. aureus* within 30 min. This light-controlled precise activation mechanism effectively reduces the background signal and improves the detection efficiency.

To further enhance the detection sensitivity, Fan et al. [122] designed a SERS detection platform for *S. aureus* detection, which combines a photoactivatable catalytic hairpin self-assembly (CHA)/Cas12a cascade system with a multifunctional core-shell DNA tetrahedron (Figure 4C). The CHA reaction is triggered in the presence of *S. aureus* by using allosteric aptamers that can specifically bind to *S. aureus*. After the CHA reaction is completed, under UV light, it can activate the trans-cleavage activity of CRISPR/Cas12a bound to the photocleavage group. After *S. aureus* triggers cleavage, the released DNA tetrahedral probe carries Raman molecules embedded in the core-shell structure (AuNPs@Fe_3_O_4_), enhancing the SERS signal through magnetic enrichment. The detection limit of this platform for *S. aureus* is 3.16 CFU/mL, which is 20 times more sensitive than traditional PCR. Moreover, the detection can be completed within 40 min, providing an efficient solution for the rapid detection of low-abundance pathogenic bacteria in clinical samples.

In the scenario of complex matrix detection, the anti-interference ability and signal self-calibration will become a difficult problem to be solved. As shown in Figure 4D, Dou et al. [123] constructed a ratio fluorescence aptamer sensor based on Zr-MOF (UiO-66-NH_2_). The MOF used in this sensor is prepared with H_2_BCTPE as the organic binder, enabling the MOF to be excited at 460 nm to produce blue-green luminescence. The AFB1 aptamer modified by Cy3 binds to NMOF through the Zr-OP bond. In the presence of AFB1, the specific binding of AFB1 and the aptamer induces conformational changes, enhancing the FRET effect and generating fluorescence signals. The detection limit of this sensor for actual corn samples can reach 0.08 ng/mL, and the detection recovery rate for actual samples can reach 89.11–102.4%. With the high stability of MOF materials and the specific recognition of aptamers, the anti-interference detection of toxins in complex food matrices was achieved.

### 3.2. Environmental Monitoring

The problem of environmental pollution is becoming increasingly serious. Conducting real-time and rapid detection of pollutants in the environment is an important link in environmental governance. Environmental pollutants include organic pollutants, biological toxins, etc. Their existence poses a serious threat to the ecological environment and human health. Traditional environmental detection methods require complex laboratory equipment and professional technicians, making it difficult to realize the on-site rapid detection of pollutants. Photoactivatable aptamers can specifically recognize target pollutants in the environment. Aptamers can be combined with the technology of POCT, which establishes rapid and sensitive detection of pollutants.

**Figure 5:**
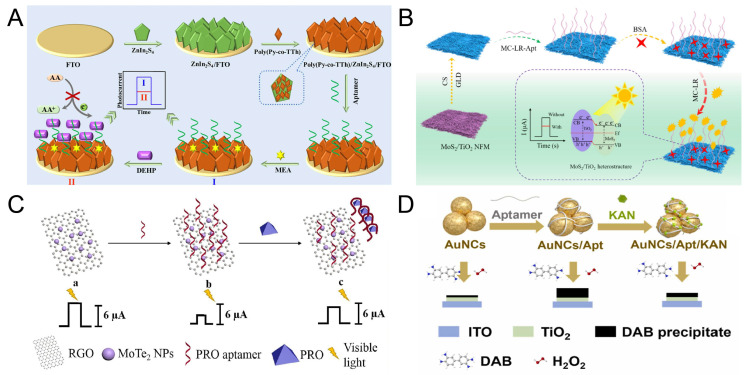
(**A**) Schematic of the near-infrared light-responsive aptamer sensor for DEHP detection. Reproduced with permission from ref. [104]. Copyright 2025, Elsevier. (**B**) The Scheme of the PEC aptasensor for MC-LR detection. Reproduced with permission from ref. [108]. Copyright 2025, Elsevier. (**C**) The Scheme of PRO aptamer sensor activated by visible light. Reproduced with permission from ref. [106]. Copyright 2021, Elsevier. (**D**) Schematic of PEGFET sensor constructed with TiO_2_ photoresponse gate and aptamer probe for detecting kanamycin. Reproduced with permission from ref. [107]. Copyright 2025, Elsevier.

Di (2-ethylhexyl) phthalate (DEHP) is often used as a plasticizer for plastic products. DEHP can easily leak from the plastic substrate into the environmental medium, thus causing great pollution to the environment. As shown in Figure 5A, Yang et al. [104] developed a NIR light-responsive aptamer sensor based on the Z-type heterojunction poly(pyrrole-co-thiophene)/ZnIn_2_S_4_ for the detection of DEHP. This heterojunction matches the energy band of ZnIn_2_S_4_ through the copolymer, forming a direct Z-type charge transfer path. The aptamer is fixed on the surface of the heterojunction through π-π stacking. After the aptamer specifically recognizes DEHP, the conformational change of the aptamer leads to an increase in the steric hindrance on the surface of the heterojunction, hindering electron transfer and causing the photocurrent signal to decrease. Under the excitation of NIR light, the efficient charge separation of Z-type heterojunction enables the detection limit of the target to be as low as 0.45 pM, and the recovery rate in lake water samples can reach 91.85–103.41%. This sensor utilizes the strong penetration characteristic of NIR light to achieve the direct detection of DEHP in turbid water bodies.

For the trace detection of microcystin toxin-LR (MC-LR), Liao et al. [108] established an aptamer sensor using MoS_2_/TiO_2_ nanofiber membranes (NFM) type II heterostructures (Figure 5B). The band offset of MoS_2_ and TiO_2_ forms Type II heterojunctions, effectively promoting the separation of photogenerated electron-hole pairs. The aptamer is fixed on the electrode surface and is used to specifically recognize MC-LR molecules. Under visible light irradiation, the combination of MC-LR and the aptamer causes the sensor to generate a photocurrent signal, and the sensor detection limit reaches 0.34 pM. This sensor realizes the portable and sensitive detection of MC-LR, and also has a good recovery rate for the detection of actual samples.

With the development of detection technology, the rapid and accurate detection of residual pesticides has become a hot topic worldwide. The increasing application of pesticides may lead to the consequence of environmental deterioration, thereby causing damage to human health. Profenofos (PRO) is a common moderately toxic organophosphate insecticide and has been classified as a Class II toxicity by the World Health Organization. As shown in Figure 5C, Wang’s team [106] developed a label-free PRO aptamer sensor using a one-step hydrothermal synthesis of molybdenum telluride/reduced graphene oxide (MoTe_2_/rGO) Schottky junction. The Schottky barrier formed by MoTe_2_ and rGO effectively inhibits the recombination of photogenerated electron-hole pairs and significantly improves the photoelectric conversion efficiency. The aptamer is fixed on the surface of MoTe_2_ through physical adsorption. In the presence of PRO, the stronger binding force between the aptamer and PRO causes the aptamer /PRO compound to be released from the electrode, reducing the resistance value. Under visible light, the detection limit of this sensor is 3.3 × 10^−10^ g/L.

Antibiotic residues in the environment may pose a serious threat to human health, so it is necessary to develop effective monitoring and control analysis strategies. As shown in Figure 5D, Ye et al. [107] proposed a PEGFET sensor based on TiO_2_ light-response gates and aptamer probes for the detection of kanamycin. This sensor uses TiO_2_ as the photosensitive layer for adsorbing UV light. The aptamer is attached to AuNCs for the recognition of kanamycin. When kanamycin is present, kanamycin molecules will bind to the surface of AuNCs through interaction with the aptamer to weaken the catalytic ability of AuNCs and increase the photocurrent. The high-sensitivity detection of kanamycin was achieved through the offset of the transmission curve and the current change after adding kanamycin. Under the condition of light exposure, the detection limit can reach 1.13 nM. Meanwhile, the detection of actual samples also proved the potential of this sensor in practical applications.

### 3.3. Biomedical Diagnostics

In the field of biomedical diagnostics, light-controlled aptamer technology is opening up new paths for the precise detection of biomarkers with its unique spatiotemporal regulation ability. Different research teams have developed a series of highly sensitive detection systems based on aptamers and light regulation, achieving certain progress in cancer diagnosis [124,125,126,127,128], organelle metabolism analysis [129,130,131,132,133,134], and intracellular molecular monitoring [135,136].

**Figure 6:**
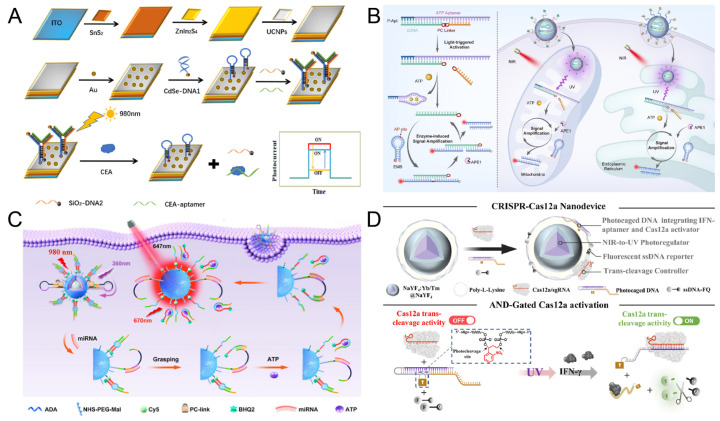
(**A**) The scheme of PEC aptamer sensor based on UCNPs and Y-type DNA. Reproduced with permission from ref. [117]. Copyright 2023, Elsevier. (**B**) Schematic of the design principle of the light and enzyme dual-regulated aptamer sensor. Reproduced with permission from ref. [114]. Copyright 2025, Wiley-VCH. (**C**) Schematic diagram of ATP and NIR photocontrolled sensors used to detect intracellular miRNAs. Reproduced with permission from ref. [110]. Copyright 2023, American Chemical Society. (**D**) Schematic of the NIR photoactivatable aptamer-CRISPR Nanodevice for monitoring IFN-γ release. Reproduced with permission from ref. [116]. Copyright 2024, American Chemical Society.

As shown in Figure 6A, Liu et al. [117] constructed a PEC aptamer sensor based on UCNPs and Y-type DNA, achieving highly sensitive detection of carcinoembryonic antigen (CEA). This sensor can regulate the dynamic switching of the signal pathway through near-infrared (NIR) light. Upconversion nanomaterials (ITO/SnS_2_/ZnIn_2_S_4_/UCNPs) were synthesized by electrostatic adsorption, and CDSe-modified DNA1 was connected to their surfaces through gold–sulfur bonds to further enhance the photoelectric conversion efficiency. The UCNPs in this nanomaterial can convert NIR light into ultraviolet-visible light, excite CdSe, and generate enhanced photocurrent. The Y-type DNA in the sensor is composed of DNA1, SiO_2_ NPS-labeled auxiliary DNA2, and CEA aptamer DNA. In the absence of a target, due to the relatively long distance between CdSe NPs and the electrode surface and the shielding effect of SiO_2_, the photocurrent decreases, resulting in the signal being turned off. When CEA is present, the aptamer specifically binds to CEA, causing conformational changes, resulting in the destruction of the Y-type structure. CdSe approaches the electrode, and the photocurrent recovers to generate a signal. By monitoring the photocurrent signal, highly sensitive detection of CEA can be achieved, with a detection limit of up to 0.3 pg/mL, and it has been successfully applied to the clinical detection of CEA in human serum samples. This light-controlled signal switching strategy provides a reliable technical means for the early diagnosis of cancer.

ATP is the main energy currency within cells, and subcellular ATP dysregulation is associated with various disease conditions. As shown in Figure 6B, Feng et al. [114] developed a DNA nanodevice based on photoactivatable aptamers. This device combines exogenous light control and endogenous enzymatic reactions, achieving precise regulation of DNA nanostructures and specific signal amplification of ATP in organelles. The ATP aptamer was hybridized with the block chain (bDNA) modified by PC-linker to form the aptamer probe. Under ultraviolet light irradiation, the PC-linker breaks, releasing the hybrid chain of the aptamer and the bDNA fragment. In the presence of ATP, the aptamer binds to ATP and releases a bDNA fragment. This fragment can hybridize with EMB modified with AP site. In the presence of APE1, AP site is cleaved, fluorescent groups and quenching groups are separated, and a fluorescence signal is generated. Meanwhile, the released bDNA fragment can initiate the next hybridization cycle. The specificity and sensitivity of ATP detection have been further enhanced, and the detection limit of ATP can reach 0.44 μM. Subsequently, UCNPs were combined with the aptamer probe and EMB and functionalized with organelle targeting ligands to form DNA nanodevices. Notably, UCNPs enable the conversion of NIR light into UV emission, thereby activating the sensing function of the aptamer probe. This strategy achieves highly selective and sensitive imaging of ATP within subcellular organelles by coupling photonic energy transfer with aptamer-target recognition dynamics.

As shown in Figure 6C, Ren et al. [110] further advanced this paradigm by integrating endogenous ATP signaling with exogenous light-controlled regulation. They engineered a bioresponsive nanomaterial that responds to intracellular ATP levels to modulate NIR-switchable fluorescence output, enabling spatiotemporally resolved detection of miRNA within living cells. This DNA nanomachine consists of three parts: double-stranded DNA (A/B), miRNA recognition hairpins modified with PC-linker (DNA-C), and UCNPs. In double-stranded DNA, fluorescent groups are modified on DNA-A for signal output, and the DNA-b sequence contains ATP aptamer sequences for ATP recognition. Under 808 nm laser irradiation, UCNPs convert NIR light into UV light to induce the cleavage of the PC-linker. Subsequently, in the presence of miR-21, DNA-C hybridizes with miR-21 and captured DNA-B. The captured DNA-B combines with ATP to release DNA-C again to form a cycle. The generation of fluorescence signals was further enhanced. The detection limit of miR-21 triggered by ATP was 5.5 pM, achieving sensitive detection of miR-21. This strategy of combining intracellular metabolites (ATP) with the detection of target miR-21 provides a new idea for the monitoring of complex intracellular molecular networks.

As shown in Figure 6D, Liu et al. [116] constructed a photoactivatable aptamer-CRISPR nanodevice for the demand of in vivo detection, which was used to precisely analyze interferon -γ (IFN-γ) released by T cells in humanized mice. This device consists of UV-cleavable PC-DNA probes embedded with aptamers, UCNPs, and a CRISPR-Cas12a-enhanced fluorescence system. Under the irradiation of 980 nm NIR light, UCNPs converts NIR light into UV light, activates the PC-DNA probe, and releases the aptamer sequence. After the aptamer specifically binds to IFN-γ, it triggers the fluorescence signal amplification mediated by CRISPR-Cas12a, achieving real-time dynamic imaging of IFN-γ. The detection limit of IFN-γ can reach 0.28 pg/mg, and it has been successfully applied to real-time dynamic imaging in a humanized mouse inflammation model. This detection method based on photoactivatable aptamers shows high sensitivity and specificity both in vivo and in vitro, and has great potential in precise in vivo analysis.

## 4. Summary and Outlook

Aptamers, with their high affinity, high specificity, and versatile modification capabilities, have become important candidates in biosensing applications. The integration of light-responsive mechanisms not only expands the modulation strategies for aptamer functionality but also significantly enhances the selectivity, sensitivity, and dynamic response capabilities of POCT systems. This article reviews the applications of photoactivatable aptamers-based POCT platforms across diverse fields. The combination of the optical response element and the aptamer realizes the precise spatiotemporal regulation and highly sensitive detection of the target molecule. The photoactivatable aptamer can significantly improve the selectivity and anti-interference ability of detection through light-induced conformational changes, charge separation or energy transfer, combined with signal amplification strategies such as CRISPR/Cas systems and RCA. At present, photoactivatable aptamers have demonstrated broad utility in multiple fields such as food safety, environmental pollution, and biomedicine. Their advantages of rapid response, low cost, and high sensitivity provide a new direction for the development of POCT technology.

Despite these advancements, practical implementation of photoactivatable aptamers faces several challenges. For instance, the dependence of some photosensitive structures on UV or visible light limits their applicability for in vivo or deep tissue detection [137,138], while their high-energy photons may also cause DNA damage and cellular phototoxicity [139]. Although the NIR response system has strong penetration, it is limited by the Stokes shift effect and the non-radiative transition mechanism [140]. Its energy conversion efficiency is generally low, and it is easily interfered by the absorption bands of hemoglobin and water molecules in deep tissues, resulting in a significant attenuation of the effective photon flux actually reaching the target [141,142,143]. Furthermore, the inconsistency of lighting conditions not only leads to signal fluctuations but also faces more complex biological constraints in live applications. Especially for clinical transformation, the existing light control devices lack unified norms in terms of irradiation area, angle and dose standardization [144]. In terms of sensor construction, although the traditional covalent coupling strategy can achieve the stable combination of photoresponsive materials and aptamers, the immobilization orientation may lead to the distortion of the three-dimensional conformation of the aptamer, significantly reducing the molecular recognition sensitivity [145]. Therefore, the coupling mode of photoresponsive materials and adaptors still needs to be further optimized to improve the response efficiency and signal-to-noise ratio.

Looking ahead, the future directions can be expanded from the following aspects to promote photoactivatable aptamer POCT. Efforts should be made to develop more efficient photoresponsive materials to address the issues of insufficient UV light penetration and light scattering interference in biological samples, while enhancing the stability and biological safety of the materials in physiological environments. An attempt has been made to build a multi-functional integrated platform, combining the light control aptamer with microfluidic chips [146], smartphone imaging, and artificial intelligence algorithms [147] to construct a fully integrated POCT platform integrating “sample processing-light control response-multi-signal output-data analysis [148]”, achieving simultaneous detection of multiple targets and real-time feedback. In addition, the standardization and clinical transformation of photoactivatable aptamer POCT are also of vital importance. It is necessary to establish a unified evaluation system for photoactivatable aptamer detection, solve the problem of non-specific adsorption in complex samples, promote the transformation of related technologies from the laboratory to commercial POCT kits, and enable them to play a role in rapid diagnosis and personalized medicine.

In conclusion, as the intersection frontier of molecular recognition and light control technology, the light-activated aptamer will continue to drive POCT to evolve towards higher sensitivity, intelligence, and scene adaptability, providing innovative tools for precision medicine and environmental health monitoring.

## Figures and Tables

**Table 1:** Summary of the optical activation aptamer sensing strategy.

Application	Target	Target Category	Detection Method	Optically Controlled Strategy	LOD	Ref.
Food safety	*S. aureus*	Bacteria	FRET, Photothermal	Polydopamine nanospheres	1.0 CFU/mL	[96]
	*S. aureus*	Bacteria	Fluorescence	PC-linker	2 CFU/mL	[97]
	*S. aureus*	Bacteria	FRET, Thermal	Electronic carbon nanoparticles	1.0 CFU/mL	[95]
	*E. coli*	Bacteria	Photothermal	CuxS nanoparticles + PC-linker	1.8 CFU/mL	[98]
	*S. typhimurium*	Bacteria	Fluorescence	Nanomaterials + Photosensitizer	2 CFU/mL	[99]
	Zearalenone ZEN	Small molecule	Photoelectrochemical	Semiconductor materials	0.087 fg/mL	[100]
	Zearalenone ZEN	Small molecule	Colorimetric	TiO_2_ NPs + PC-linker	0.0087 ng/mL	[101]
	Aflatoxin	Small molecule	Fluorescence	PC-linker	0.074 ng/mL	[83]
	Tetracycline	Small molecule	Photoelectrochemical	Semiconductor materials	1.6 nM	[102]
Environmental monitoring	Chloramphenicol	Small molecule	Electrochemical	Semiconductor materials	0.17 pM	[103]
	Di (2-ethylhexyl) phthalate	Small molecule	Photoelectrochemical	Semiconductor materials	0.45 pM	[104]
	Enrofloxacin	Small molecule	Photoelectrochemical	CN QDs	0.033 ng/mL	[105]
	Profenofos	Small molecule	Photoelectrochemical	Semiconductor materials	0.33 pg·mL	[106]
	Omethoate	Small molecule	Photoelectrochemical	Photoactive nanomaterial	0.0027 nM	[90]
	Kanamycin	Small molecule	Photoelectrochemical	Photoactive nanomaterial	1.13 nM	[107]
	Microcystin toxin-LR	Small molecule	Photoelectrochemical	Semiconductor materials	0.34 pM	[108]
Biomedical diagnostics	let-7a	microRNA	Fluorescence	PC-linker	1.0 pM	[109]
	miRNA-21	microRNA	Fluorescence	UCNPs + PC-linker	18 pM	[110]
	miRNA-21	microRNA	Fluorescence	PC-linker	0.025 nM	[111]
	ASFV p72 gene	Virus	Fluorescence	PC-linker	2.5 copies/µL	[112]
	ATP	Small molecule	Electrochemical	Electrode material	0.5 nM	[113]
	ATP	Small molecule	Fluorescence	PC-linker	3.7 μM	[52]
	ATP	Small molecule	Fluorescence	UCNPs + PC-linker	0.44 μM	[114]
	Cocaine	Small molecule	Colorimetric	Photoactivated oxidases	0.38 μM	[115]
	IFN-γ	Cytokine	Fluorescence	UCNPs + PC-linker	0.45 pg/mg	[116]
	Estradiol	Small molecule	Colorimetric	Photoactivated oxidases	326 nM	[87]
	Carcinoembryonic antigen	Protein	Photoelectrochemical	Semiconductor materials	0.3 pg/L	[117]
	Thrombin	Protein	Fluorescence	FRET	112 nM	[118]
